# Educational inequalities in aging-related declines in fluid cognition and the onset of cognitive pathology

**DOI:** 10.1016/j.dadm.2015.06.001

**Published:** 2015-06-28

**Authors:** Sean A.P. Clouston, M. Maria Glymour, Graciela Muñiz Terrera

**Affiliations:** a Program in Public Health and Department of Preventive Medicine, Stony Brook University, Stony Brook, NY, USA; b Department of Epidemiology & Biostatistics, University of California, San Francisco, CA, USA; c MRC Unit for Lifelong Health and Ageing, University College London, London, UK

**Keywords:** Dementia, Aging, Neurology, Social medicine, Cognitive reserve, Educational status

## Abstract

**Background:**

Education has been robustly associated with cognitive reserve and dementia, but not with the rate of cognitive aging, resulting in some confusion about the mechanisms of cognitive aging. This study uses longitudinal data to differentiate between trajectories indicative of healthy versus pathologic cognitive aging.

**Methods:**

Participants included 9401 Health and Retirement Study respondents aged ≥55 years who completed cognitive testing regularly over 17.3 years until most recently in 2012. Individual-specific random change-point modeling was used to identify age of incident pathologic decline; acceleration is interpreted as indicating likely onset of pathologic decline when it is significant and negative.

**Results:**

These methods detect incident dementia diagnoses with specificity/sensitivity of 89.3%/44.3%, 5.6 years before diagnosis. Each year of education was associated with 0.09 (95% confidence interval [CI], 0.087–0.096; *P* < .001) standard deviation higher baseline cognition and delayed onset of cognitive pathology (hazard ratio, 0.98; 95% CI, 0.96–0.99; *P* = .006).

**Conclusions:**

Longitudinal random change-point modeling was able to reliably identify incident dementia. Accounting for incident cognitive pathology, we find that education predicts cognitive capability and delayed onset pathologic declines.

## 1 Introduction

Dementia affects as many as 5 million Americans living with the disease and 15.5 million engaged in caring for friends or family [1]. Dementia is characterized by rapid changes in individual capabilities and behaviors [2] but most commonly affects domains of “fluid cognition” including memory, executive functioning, fluency, and mental status [3]. Dementia is believed to be the end result of progressive neuropathologic changes, with most individuals experiencing milder forms of cognitive impairment before ultimately meeting diagnostic criteria [4].

Education has been shown to be a robust predictor of a broad range of health indicators [5], including dementia [6], [7]. Sociologic theory suggests that social factors should influence healthy aging because they determine access to resources, including for instance knowledge or money, that are known to broadly influence health and disease [8], [9]. Education is further associated with “cognitive reserve,” the brain's ability to maintain healthy functioning into old age despite increasing neuropathology [10], which is believed to be attained in part through an association with higher cognitive capability [11], [12] and associated brain functional efficiency [10]. Moreover, education modifies access to many resources commonly implicated in maintenance of cognitive reserve over an entire life course [3], [13] such as opportunities for social and cognitive engagement [14], [15] and the risk of physical pathology [9], [16], [17].

Although researchers suggest that education is associated with improved cognition and reduced risk of cognitive impairment and dementia [11], [12], [18], longitudinal associations between education and rate of cognitive aging have been inconsistent [18], [19], [20], [21], [22]. Life course epidemiologists have recently suggested that “healthy” aging may be differentiated from pathologic aging, which could be described as a pattern of more rapid aging and the onset of disease [23]. Although modest cognitive declines may occur even in healthy aging, this article posits that rapid acceleration of cognitive decline may reflect neuropathologic changes that can be used to differentiate between healthy aging and onset of pathologic aging [20], [24].

### 1.1 Hypotheses

**Fig. 1:**
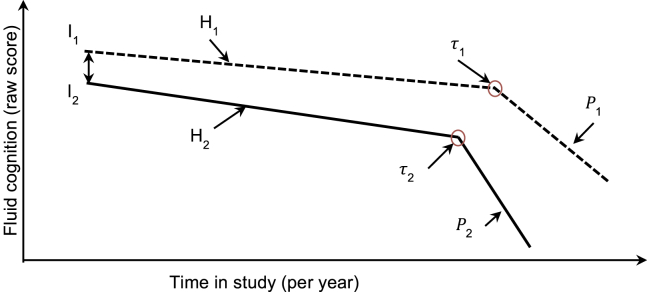
Graphical hypotheses linking education to cognitive aging. For each individual (i), the rate of healthy declines (H_i_), baseline differences (I_i_), rate of pathologic decline (P_i_), and age of onset (*τ*_*i*_) is indicated.

We hypothesize (see Fig. 1 for graphical hypotheses) that education will be associated with differences in baseline capability (hypothesis 1: I_1_ ≠ I_2_), the rapidity of healthy declines (hypothesis 2: H_1_ ≠ H_2_), hazard of onset of pathologic declines (hypothesis 3: *τ*_1_ ≠ *τ*_2_), and the rapidity of pathologic declines after onset (hypothesis 4: *P*_1_ ≠ *P*_2_).

## 2 Data

We use waves 3-10 of the Health and Retirement Study (HRS), which has collected cognitive data biennially from 1996 to 2012 (response rate 81.6%) and is publically available online (http://hrsonline.isr.umich.edu) [25]. Since 1996, the HRS has increased enrollment; we have included all new enrollees. Of 26,048 respondents with at least one valid cognitive observation, we limited analysis to 25,957 respondents with valid educational information. Finally, pathology identification routines required that respondents have at least five waves of data for identification. Our analytic sample therefore included 9401 respondents who were observed a total of 58,640 times for between 7.1 and 17.3 years (Table 1).

## 3 Measures

Fluid cognition encapsulates cognitive domains such as memory or executive functioning that are most susceptible to cognitive pathology [26]. Fluid cognition is often measured using a modified version of the telephone interview for cognitive status [27], [28]. In the HRS, cognition was measured using a composite index (/35 points) made of commonly used cognitive tests derived from the telephone interview of cognitive status [29]. Verbal learning asks individuals to learn a list of 10 words and repeat as many back to the interviewer correctly as possible (/10). Verbal memory asks respondents to again recall the list of words around 12 minutes later, after intermediary questioning (/10). Working memory was measured using the serial 7s subtraction test, which asks individuals to subtract 7 sequentially starting at 100 and continuing for five trials (/5). Backward counting asks individuals to count backward from a specified number as quickly as possible (/2). Presidents ask respondents to name the current president/vice-president (/2). Object naming asks individuals to correctly name two objects provided verbal descriptions (/2). Orientation asks respondents to correctly note the date (day, month, year) and day of the week (/4). For ease of comparison, we standardized fluid cognition. Because many individuals are lost to follow-up due in part to poor cognition [30], we used information provided by the HRS that incorporates imputed scores derived from the information provided by proxy respondents to substantially reduce attrition bias [31].

Probable cognitive pathology is indicated using profile likelihood estimation [14], which maximizes the log-likelihood to identify the age at which pathologic acceleration began (hereafter “onset”). This identifies a potential change-point for every respondent; however, in this sample it is unlikely that all respondents will experience the onset of pathology during observation. Akaike's information criteria (AIC) were used to compare pathologic and linear models for each individual. Because profile likelihood is sensitive to random variation in the first or last waves, we ignored onset that was indicated to occur before the second or after the penultimate waves. Pathology onset was recorded for 72.9% of respondents whose change-point model was deemed preferable to the linear one. Onset was further ignored for 33.3% of the remaining sample, whose post-onset slopes indicated positive acceleration. For specificity analyses, we also make use of self-reported Alzheimer's disease or dementia diagnoses in the last two waves of observation.

Education was measured at baseline in years of education and ranged from 0 to 17 in these data. It is standard in epidemiologic analysis to adjust for age and sex; we measured age in months/12 at baseline (centered at age 65) and indicate female sex. To model change over time, we use measurement wave, which indicates the time, measured in months, but specified in years as (month–0.5)/12 after the first measurement wave; observations in the subsequent waves occurred between 1.17 and 3.25 years from baseline observation.

## 4 Methods

Longitudinal multilevel modeling (MLM) was used to model cognitive aging. We follow prior analysts [32] who use MLM, assuming that observations are autocorrelated within-individuals MLM separately estimates individual-level random “intercepts” and “slopes” following equation 1:

(1) $$Y_{it}=\beta_{0}+\beta_{1}F+\beta_{2}A_{0}+\beta_{3}E_{i}+\gamma_{0i}+\left(\beta_{4}+\beta_{5}E_{i}+\gamma_{1i}\right)t_{it}+\left(\beta_{6}+\beta_{7}E_{i}+\gamma_{2i}\right){\left(t_{it}-\tau_{i}\right)}^{+}+\varepsilon_{it}$$

where *Y*_*it*_ is the expected cognitive performance for individual “*i*” at time “*t*,” ${\left(t_{it}-\tau_{i}\right)}^{+}$ represents the accelerated slope noting that *f(x)^+^ = f(x)* when *f(x)* > 0 and 0 otherwise, *τ*_*i*_ represents the individual's follow-up time before onset of pathologic declines, *A*_*0*_ is age at baseline, *S* indicates respondent sex, *E*_*i*_ is education in years, and *γ*_*0i*_, *γ*_*1i*_, and *γ*_*2i*_ refer to random intercepts, healthy slopes, and pathologic slopes, respectively. We calculated life expectancy free of cognitive pathology: $\tau_{i}=\frac{e^{-rA_{0}}}{r}$, where *r* is the incidence rate calculated using hazards regression (Appendix A). To calculate the incidence rate (*r*), using estimates derived from Cox proportional hazards regression [33] (results are provided in the following): $\ln\left(\frac{H\left(\tau_{i}\right)}{H\left(\tau_{0}\right)}\right)=\theta_{1}S+\theta_{2}E_{i}\text{,}$ where *θ* are coefficients estimated when predicting the hazard function [*H(t)*]. We assume that respondents are free of cognitive pathology at baseline. Schoenfeld residuals were used to examine the proportional hazards assumption. These analyses had 80% power, to detect a hazard ratio (HR) = 0.84 (α = 0.05). Because longitudinal models rely on the assumption that pathologic slopes are theoretically meaningful, we provide the sensitivity and specificity of our measure of cognitive pathology to identify newly reported diagnoses of dementia and Alzheimer's disease in the final wave of follow-up; we also provide odds ratios (OR) estimated using logistic regression to examine predictive power after adjusting for age at baseline.

In longitudinal models, the influence of education is explicitly modeled as modifying intercepts (*β*_*3*_), slopes (*β*_*5*_), acceleration (*β*_*7*_), and survival time (*θ*_*2*_). Longitudinal MLM is useful in part because it robustly accounts for repeat-observation biases and heteroskedasticity in slopes over time. We assume an unstructured covariance matrix and further estimate the covariance between intercepts and slopes. We assume that errors are distributed multivariate normal. We model linear declines before and after the change-point. To examine model fit, we report adjusted pseudo-*R*^2^ and model-specific AIC; we further provide the difference between AICs (ΔAIC) to explicitly compare two models. We provide standardized β coefficients, standard errors, and exact *P* values. Stata version 13.1/IC was used for analyses. The Stony Brook University Ethics Board declared this study not human subjects research.

### 4.1 Sensitivity analyses

Using longitudinal modeling is the gold standard in cognitive aging studies; however, longitudinal modeling may be mis-specified if “healthy aging” occurs more slowly than “pathologic aging” and pathologic aging occurs nearer the end of the observation: pathologic observations, being more extreme and lower than other observations, will negatively bias within-person slopes. Sensitivity analyses were conducted to assess the relative model fit of models estimating pathologic decline versus those using quadratic or linear slopes.

## 5 Role of the funding source

The funder played no role in data collection, analysis, interpretation, reporting, or the decision to submit the article for publication. The corresponding author had full access to the data and has responsibility for the decision to submit for publication.

## 6 Results

### 6.1 Sample characteristics

Baseline sample characteristics (Table 1) show that the sample is predominantly female and most completed at least a high school education. The average observed age at onset was 75.4 ± 6.77 years. Those who experienced onset were 2.9 years older at baseline (t = 15.53, *P* < .001), had lower education (t = 4.11, *P* < .001), and lower baseline fluid cognition (t = 7.82, *P* < .001). The overall incidence rate was 1.63% (1.56–1.71) and correlated with years of education (*r* = −0.51).

On average, onset of cognitive pathology occurred 5.6 years younger than new diagnosis with dementia. Notably, those with non-trivial pathologic change-points were more likely to report new diagnoses of dementia (OR = 2.98, [1.49–5.94], *P* = .002) or Alzheimer's disease (OR = 3.42, [1.87–6.24], *P* < .001), conditional on lack of prior diagnoses of dementia. We were able to predict newly diagnosed Alzheimer's disease and dementia in the final wave with a specificity/sensitivity of 86.3%/41.7% and 89.3%/44.3%, respectively.

### 6.2 Hazards of pathologic decline

**Fig. 2:**
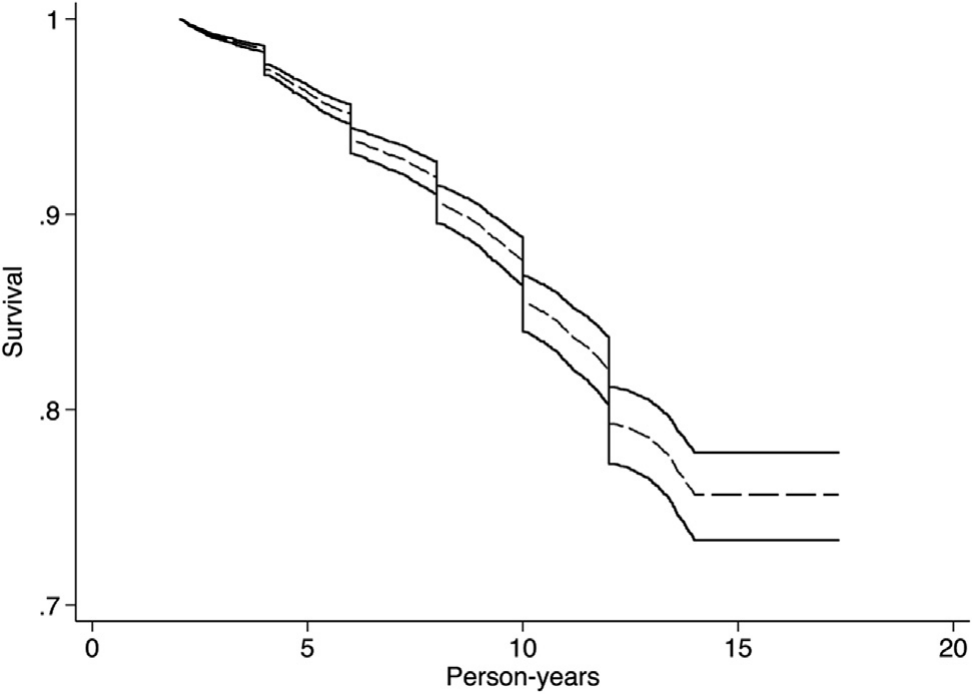
Survival from the onset of cognitive pathology. Survival curves are separated into those with 7 years of education (solid), 12 years of education (dashed), and 17 years of education (dotted). Health and Retirement Study 1996–2012.

Examining the hazards of the onset of pathology (Fig. 2), we found that sex was associated with the risk of cognitive decline (HR = 0.89; 95% confidence interval [CI], 0.81–0.98; *P* = .015). Furthermore, each year of schooling was associated with lower risk of cognitive pathology (HR = 0.98; 95% CI = [0.96–0.99]; *P* = .006). Age-specific prevalence of cognitive pathology in this sample is 1.33%, 2.84%, 3.92%, 5.73%, 6.61%, and 7.58% for ages 65–69, 70–74, 75–79, 80–84, 85–89, and 90–94, respectively.

### 6.3 Longitudinal model of change

Education was strongly associated with cognitive capability at baseline (Table 2). Specifically, having more years of education was associated with higher baseline capability. Incorporating the interaction between education and healthy or pathologic aging did not improve model fit (Table S1). Notably in these models, random-effect estimates show a weak association between random intercepts and healthy slopes, a moderate association between random healthy and pathologic slopes, and no association between random intercepts and random pathologic slopes.

### 6.4 Sensitivity analyses

Comparing pathologic change models to other comparable models (Table S2), we note that the model provided here outperforms other models of acceleration. Results from these models show that within-person estimates of decline were three times more rapid using linear slopes (model 3; β = −0.059), than they were when using random quadratic slopes (model 5; β = −0.021). Modeling pathologic slopes result in slightly slower healthy slope estimates (model 7, β = −0.019), suggesting that healthy aging occurs less rapidly than often estimated. The best fitting model is one that models random healthy and pathologic slopes.

## 7 Discussion

Social epidemiology suggests that education should influence the rapidity of cognitive aging, although results have been inconsistent. We differentiate “healthy” from “pathologic” aging to examine the role of education on cognitive aging and find preliminary support for the view that education is associated with cognitive aging. Specifically, we found some support for the documented association between education and baseline capability and further found a strong association with delayed onset of cognitive pathology. These results support two of our hypothesized theories and highlight one way to differentiate types of aging in future research.

### 7.1 Social epidemiology

Prior research has noted that education is robustly associated with baseline cognition [11], [19], a finding that is replicated here. Furthermore, results support previous studies noting that education does little to influence cognitive aging [22], [34], [35]. Yet, our research also argues that education helps to delay the onset of cognitive pathology. We found that education was associated with delayed onset of pathologic declines although education was not as robustly associated with rates of either healthy or pathologic aging. Our analyses suggest that modeling random acceleration improves model fit. However, further research should replicate, simulate, and extend these findings into other contexts and samples.

Results support theories of cognitive reserve [10] and life course theories of cognitive aging [11], [36], suggesting that early life investments reap rewards in late-life. However, analyses suggest that the educational benefit may have a threshold. Socioeconomic resources are believed to broadly influence the “risk of risks” [37], and it may be that the mechanisms linking education to cognitive aging are more influential at the lower range of the spectrum. There are a number of known risk factors for dementia that may be associated with socioeconomic status including the risk of cardiovascular disease [38] and decreased engagement in activities that may improve cognitive aging [39]. Furthermore, educational attainment predicts risk of poor health behaviors [40], which have long been associated with shorter lives and poorer health outcomes across a broad range of physical and cognitive capabilities later in life [41]. Finally, education is a persistent predictor of the risk of all cause mortality [16] and results may reflect delayed terminal cognitive decline [42].

### 7.2 Healthy versus pathologic aging

Results underscore the importance of differentiating healthy from pathologic aging in studies of cognitive aging. This may be useful to epidemiologists interested in examining the predictors of “healthy” aging [8]. If “healthy aging” occurs more slowly while “pathologic aging” occurs more rapidly and nearer the end of observation [43], these models may also reduce the bias associated with accelerated cognitive aging associated with incident dementia or terminal decline. Notably, these results suggest that pathologic aging may be a within-person process.

### 7.3 Measures of pathology

Differentiating between healthy and pathologic declines provides a foundation on which to robustly integrate longitudinal analyses of cognitive aging with analyses of dementia. Separating healthy aging from brain pathology is not a novel endeavor [44]. Within the study of cognitive aging, researchers have separately examined terminal declines [42] and accelerated changes before dementia diagnoses [45]. Given that late-life deterioration likely results from interactions among aging and disease, these effects may not be fully distinguishable [46], [47]. We uniquely used inferential statistics to identify when individuals likely began experiencing pathologic declines under the assumption that aging accelerates among those who will experience dementia [4]. We found that onset in this study predated newly reported dementia diagnosis and that we were reliably able to identify those who self-report being diagnosed with dementia with sensitivity/specificity of 44.3%/89.3%, respectively.

It is unlikely that this method will ever be a stand-alone diagnostic test but may provide a useful way to monitor and identify patients who are likely experiencing pathologic decline indicative of the potential for incident dementia. Yet, such identification may further help us to conceptualize the rate of “normal” aging. In our data, there were a number of patterns of “aging” that ultimately reported being diagnosed with dementia, the pattern shown here, as well as a linear stable pattern of low functioning. It is unlikely that such low functioning, which will screen positive for testing, is actually a progressive disease and may instead represent low capability rather than dementia. Such misdiagnoses may be problematic for research hoping to examine conversion from milder forms of cognitive impairment to dementia because those with consistently low cognitive capability may screen positive for milder forms of cognitive impairment but may not be at as great a risk of conversion. Crucially, although dementia is believed to be generally underdiagnosed [48], [49], and by as much as 50%–80% worldwide [50], this method identified when individuals began experiencing early cognitive pathology and may therefore offer an opportunity for earlier detection and intervention of cognitive pathology.

### 7.4 Limitations

These analyses are limited by having data collected biennially and more frequent cognitive surveillance could provide more sensitive model specifications. A small number of respondents appear to have experienced the onset of pathology before observation, reducing specificity. Requiring that respondents were observed at least five times before being included in this study and were “at risk” of developing pathologic declines resulted in the exclusion of a number of respondents, reducing generalizability. In particular, our sample is marginally younger (0.2 years) and contains slightly more females (by 2.5%) than the HRS as a whole. Future analyses may seek to exclude individuals based on independent testing that seeks to identify cognitive impairment separate from modeling concerns.

Although cognitive capability is robustly associated with education [11], [12], we could not adjust for childhood cognition [3]. Analyses using life course data sets could examine the role of childhood cognition; however, to date, these data sets do not incorporate sufficient numbers of waves to facilitate using these methods.

Educational attainment has often been characterized as qualifications earned rather than years of education. We analyzed both and found results did not depend on specification and specification did little to modify model fit. However, years of education facilitated more streamlined discussion and we thus reported these results here.

Clinical information was lacking from these data, resulting in a number of limitations. We could not assess whether pathologic onset was representative of, or sensitive to, specific types of pathology. The monitoring test used, although being a common neuropsychological test in telephone surveys, is not as powerful or precise as, for example, computer-administered measures. Furthermore, we relied on self-reported diagnoses of dementia, which are known to be under-reported by 50-80% in the general population [50]. Specificity may be limited by episodic reductions in capability if respondents experienced rapid declines during observation and, unbeknownst to us, improved afterward. We reduced the likelihood of episodic bias by requiring two observations after a change-point; however, more frequent repeated measures could also provide more conclusive identification. Importantly, if dementia is underdiagnosed in these data, then our estimate of the sensitivity of these analyses will be similarly underestimated.

Although our measure of pathology strongly correlates with newly diagnosed dementia in the final wave, our sensitivity was hampered by a large number of individuals identified where diagnosis was not reported. Importantly, if dementia is under-diagnosed in these data, then our estimate of the sensitivity of these analyses will be similarly underestimated. Unlike most forms of diagnostic testing, lowered sensitivity may reflect delays between pathologic onset and self-reported clinical indications of dementia. Indeed, some of those who were identified as having dementia exhibited decline similar to pathologic rather than healthy declines throughout this period: assuming that no acceleration in decline occurred and linearly interpolating until age 25 estimates capability to be approximately 10 standard deviations higher than the population average.

Although these are substantial limitations, this study is innovative in suggesting that analyses of cognitive aging could substantially benefit from explicitly differentiating healthy aging from cognitive pathology. Furthermore, it is unique in showing robust relationships between education and the onset and rapidity of pathologic cognitive aging. It uses innovative measurement techniques to find a robust relationship between education and the onset of cognitive pathology. Finally, this study makes inroads into broadly examining social determinants of cognitive aging.

**Research in context**

1. Systematic Review: Educational attainment has been suggested to prevent dementia but is not associated with rates of decline, a conundrum that is problematic for dementia research. Dementia is marked by a relatively rapid progressive reduction in fluid cognitive capabilities in the years before diagnosis. Current longitudinal research on cognitive aging has largely assumed a linear rate of decline. There are no biomarkers for dementia that can accurately and objectively identify those who have or will get dementia.
2. Interpretation: This article uses longitudinal data to model a nonlinear pattern of aging that is suggestive of dementia. We find that we are able to use longitudinal data to objectively identify probable cognitive pathology, which can be used to indicate self-reported incident dementia (sensitivity/specificity = 44%/89%), precedes dementia diagnoses by approximately 6 years. Longitudinal modeling further finds that educational attainment predicts a delay in incident cognitive pathology.
3. Future Directions: Understanding predictors of cognitive pathology may provide a better understanding of the disease process. Examining better ways to objectively identify dementia may improve efforts to research and treat the disease.

## Figures and Tables

**Table 1:** Baseline characteristics, including percentages, means, and standard deviations, for the whole sample and separated by observed pathologic status, Health and Retirement Study 1996–2012

Characteristics	Overall		Healthy aging		Sample who experienced onset	
Mean	SD	Mean	SD	Mean	SD
Fluid cognition	24.08	4.14	24.26	4.15	23.67	3.83
Education per year	12.64	2.76	12.68	2.75	12.51	2.82
Age in years	66.63	7.46	66.02	7.42	68.91	7.18

	%	%	%
Female	58.82	58.01	61.88
n	9401	7309	1944

Abbreviation: SD, standard deviation.

**Table 2:** Standardized β coefficients and random-effect parameters examining the longitudinal association between education and both healthy and pathologic cognitive aging, Health and Retirement Study 1996–2012

Parameters	β	SE	*P*
Time	−0.032	0.001	<.001
Acceleration	−0.023	0.001	<.001
Age at baseline	−0.200	0.012	<.001
Male	Reference		
Female	−0.060	0.002	<.001
Years of education	0.092	0.002	<.001
Constant	−0.597	0.029	<.001
SD(healthy slope)	0.032	0.003	
SD(pathologic slope)	0.061	0.005	
SD(intercept)	0.509	0.007	
Corr(pathologic, healthy slopes)	−0.002	0.166	
Corr(healthy slope, intercept)	−0.166	0.051	
Corr(pathologic slope, intercept)	0.006	0.048	
SD(residual)	0.556	0.002	
Survival model			
Years of education	−0.024	0.008	.002
Male	Reference		
Female	0.113	0.042	.015

Abbreviations: SE, standard error; SD, standard deviation.

Note. SD(x) provides an estimate of the standard deviation of x. Corr(x_1_, x_2_) provides correlation between random-effect estimates. Pseudo-*R*^2^ is 0.233, *P* < .001.
