# Supplementary material for: Educational inequalities in aging-related declines in fluid cognition and the onset of cognitive pathology
Source: Alzheimers Dement (Amst). 2015 Jun 28;1(3):303–10. doi: 10.1016/j.dadm.2015.06.001 (PMC4542007; doi:10.1016/j.dadm.2015.06.001)
Supplement: Appendix A [file mmc1.pdf]

Table S1. Estimates of model fit and estimates for the relative benefit of adding in education, Health and Retirement Study 1996-2012

	Model 1	Model 2	Model 3	Model 4
	Baseline	Education intercepts	Education x Slope	Education x Pathological slope
AIC	119194	117411	117318	117318
ΔAIC		-1783	-93	0
Pseudo-R^2	0.232	0.244	0.244	0.244
ΔPseudo-R^2		0.011	0.001	0.000
P	<0.001	<0.001	0.114	0.300

Note: AIC: Akaike's information criteria. ΔAIC: Difference in AIC between model n and model n-1. Pseudo-R^2 gives McFadden's adjusted Pseudo-R^2 statistic. ΔPseudo-R^2 measures difference between Pseudo-R^2 for model n and model n-1. P-values are derived from F-tests and test whether ΔPseudo-R^2 is significantly different from zero. The above fit analyses come from equation 1 in the text. Model 1 incorporates only β_0, β_1, β_2, β_4, β_6, γ_{0i}, γ_{1i}, & γ_{2i}. Model 2 additionally adjusts for education (β_3), model 3 adjusts for education x healthy slope (β_5), and model 4 adjusts for education x pathological slope (β_7).

Table S2. Fit analyses comparing baseline fit of linear versus quadratic and accelerated models, Health and Retirement Study 1996-2012

	Model 1	Model 2	Model 3	Model 4	Model 5	Model 6	Model 7
	Age	Fixed Linear Slopes	Random Linear Slopes	Fixed Quadratic Slopes	Random Quadratic Slopes	Fixed Pathological Slopes	Random Pathological Slopes
AIC	132060	123324	121252	120706	120648	119668	119310
ΔAIC		-8736	-2072	-546	-57	-980	-358
Pseudo-R^2	0.149	0.206	0.219	0.222	0.223	0.229	0.231
ΔPseudo-R^2		0.056	0.013	0.004	0.000	0.006	0.002
P	<0.001	<0.001	<0.001	<0.001	0.122	<0.001	<0.001

Note: AIC: Akaike's information criteria. ΔAIC: Difference in AIC between model n and model n-1. Pseudo-R^2 gives McFadden's adjusted Pseudo-R^2 statistic. ΔPseudo-R^2 measures difference between Pseudo-R^2 for model n and model n-1. P-values are derived from F-tests and test whether ΔPseudo-R^2 is significantly different from zero. The above fit analyses come from the following model: $Y_{it} = \beta_0 A_0 + \gamma_{0i} + \beta_1 t + \gamma_{1i} t + \beta_2 t^2 + \gamma_{2i} t^2 + \beta_3 (t - \tau_i)^+ + \gamma_{3i} (t - \tau_i)^+$ where model 1 incorporates only β_0 and γ_{0i}, model 2 adds β_1, model 3 adds γ_{1i}, model 4 adds β_2, model 5 adds γ_{2i}, model 6 replaces β_2 with β_3 and drops γ_{2i}, and model 7 adds γ_{3i}.

Appendix A. Proof of derived rate of decline.

Survival can be modeled in a number of ways. In general, exponential decay occurs if it decreases at a rate that is proportional to some value at baseline. One way to specify it is:

1) $A(t) = A_0 e^{-rt}$

where *P(t)* is the expected surviving until time t, P_0 is the likelihood of having survived until time 0, and *r* is the failure rate. In a continuous sample, we must simply integrate the curve to calculate healthy life expectancy and evaluate it at the correct points. The integration proceeds:

2) $\int_{t=0}^{\infty} A(t)dt = \int_{t=0}^{\infty} A_0 e^{-rt} dt$

Integration provides us with the following solution:

3) $\frac{A_0}{-r} e^{-rt} \Big|_{t=0}^{\infty}$

However, there are some details. First, we are mostly interested in estimating the expected point of pathological onset noting that all individuals are assumed to be disease free at baseline, implying that A_0 is 1. Second, t=0 is not, in this case, specified when individuals are born (a true t=0) but is instead specified when individuals present at baseline (t_0). Thus, evaluating the integral gives us:

4) $\frac{e^{-r\infty}}{-r} - \frac{e^{-rt_0}}{-r}$

The limit of the first term as t → ∞ is zero, which leaves us with an analytic solution that is easy to calculate for each individual in the sample using their baseline age and an incidence rate calculated from the survival model provided.

Note: for this solution to be correct, we must assume that people present at baseline are free of cognitive pathology.
